# Co-Digestion of Sugar Beet Silage Increases Biogas Yield from Fibrous Substrates

**DOI:** 10.1155/2016/2147513

**Published:** 2016-10-11

**Authors:** Sharif Ahmed, Daniel Einfalt, Marian Kazda

**Affiliations:** Institute of Systematic Botany and Ecology, Ulm University, Albert-Einstein-Allee 11, 89081 Ulm, Germany

## Abstract

This study tested the hypothesis that the easily degradable carbohydrates of the sugar beet silage (S) will improve the anaerobic digestion of grass silage (G) more profoundly compared to co-digestion of sugar beet silage with maize silage (M). M : S and G : S mixtures were tested in two continuous laboratory-scale AD experiments at volatile solid ratios of 1 : 0, 6 : 1, 3 : 1, and 1 : 3 at organic loading rates of 1.5 kgVS m^−3^ day^−1^. While the sugar beet effects in mixtures with maize silage were negligible, co-digestion with grass silage showed a beneficial performance. There, the specific methane production rate was 0.27 l_N_ kg^−1^VS h^−1^at G : S ratio of 6 : 1 compared to G : S 1 : 0 with 0.14 l_N_ kg^−1^VS h^−1^. In comparison to G : S 1 : 0, about 44% and 62% higher biogas yields were obtained at G : S 6 : 1 and 3 : 1, respectively. Also, the highest methane concentration was found in G : S at ratio of 1 : 3. Synergistic increase of methane yield was found in co-digestion in both experiments, but higher effect was realized in G : S, independently of the amount of sugar beet silage. The findings of this study emphasize the improvement of AD of grass silage by even low addition of sugar beet silage.

## 1. Introduction

According to the NREAPs (national renewable energy action plans), reported by European commission, 45% of total renewable energy production shall be covered by biomass (like manure, sewage sludge, residues, energy crops, etc.) by 2020 [1]. Among different energy crops, maize silage and grass silage are considered to be the most promising biomass sources as they can generate high energy yield [2]. Hence, both substrates play an important role in the anaerobic digestion process for biogas production. In Europe, Germany is the largest biogas producer and energy crops contribute about 52% of the total substrate input. Among energy crops, maize silage and grass silage take a share of 73% and 12%, respectively [3].

Monofermentation of agricultural crops can be seen as a difficult anaerobic fermentation. It can lead to technical and biological problems within the biogas process. Such problems can be insufficient hydraulic retention time [4, 5], acidification due to inadequate buffer capacities, or nutrient deficiencies, all resulting in low methane yields [6, 7]. Fibre-rich feedstocks like grass silage, materials from public and private gardens or landscaping, are often left aside due to low biogas yields and process difficulties [8]. On the other hand, grass silage is known as a feedstock of low degradability due to its low water soluble carbohydrates and high proportion of proteins [9]. It contains high amounts of lignin but also of nitrogen and thus increasing ammonia concentration may inhibit methane formation in monofermentation [10]. Low buffering capacity during monofermentation of grass silage led to the necessity of adding alkalinity in order to stabilize the process [11].

Most of the agricultural biogas plants are using continuously stirred tank reactor (CSTR) in early years mainly designed as one-stage anaerobic digestion (AD) process. Later, biogas producing techniques using two-stage AD processes [12, 13] and leach bed reactor [14–16] were investigated to mitigate above mentioned problems by energy crops. Lindner et al. [12] claimed that two-stage AD process was appropriate for sugar rich substrate like sugar beet silage. Nizami and Murphy [15] obtained 341 l_N_ CH_4_ kg^−1^VS from grass silage using leach bed reactors. However, various drawbacks were observed such as acid production resulting in fluctuation of gas production in leach bed reactor [12, 17] and operational costs are increasing with two-stage AD process. Hence, these techniques are still challenging in terms of large scale biogas plants with continuous operation mode.

However, co-digestion of several substrates has advantages in terms of positive synergisms and supplies nutrient to microbial communities [18–20]. Animal manure is mostly used as a co-feedstock together with agricultural crops for biogas production. It provides nutrients, has good buffering capacity, and balances the carbon to nitrogen ratio due to admixing plant material high in carbon, thus preventing ammonia inhibition [21–23]. Manure addition to agricultural crops like maize silage [24], grass silage [25], and sugar beet silage [26] was already thoroughly investigated and showed generally positive effects on the biogas process.

The availability of manure can be locally limited with increasing demand. Limitation of manure can be expected as stock farming is declining [27]. Demirel and Scherer [28] reported that about 15% of biogas plants in Germany are operated without manure addition because of logistics problems. This shows that, besides the known positive effects of manure in co-digestion, there is a necessity to investigate ways of how to increase the digestibility of fibrous substrates like grass silage. In this regard, sugar beet silage can be beneficial as it contains easily degradable substrates. According to Scherer et al. [23], sugar beet silage with alkalinity range (2.5–6 g CaCO_3_-equivalents L^−1^) can be used in combination with another low-buffered crop like maize in anaerobic digestion.

Also, monofermentation of sugar beet silage cannot be recommended. Sugar beet silage shows low pH values (3.3 to 3.5) and low buffering capacities. Additionally, sugar beet silage contains also low nitrogen and phosphorus concentrations. It contains sugars and alcohols which degrade rapidly. This can lead to acidification of reactor slurry and consequently inhibiting methane production [29]. In AD experiments, utilizing sugar beet silage as single feedstock, buffering agents were added regularly in order to keep the reactor pH stable [30]. Its monofermentation can also cause foam formation in the anaerobic digester [31]. Various operational problems like plugging of gas pipes, dead zones created by inversion of digester solid profiles, or structural damage to the digester roof occurred due to foam formation [32]. However, it showed that anaerobic co-fermentation of sugar beet silage with low-buffered feedstocks like maize silage or grass silage avoided foam formation but not in the case when manure was added to the feedstock [31].

Co-fermentation of sugar beet silage with fibrous substrates like maize silage was already investigated in some studies but rarely examined together with grass silage. Nges et al. [5] found in laboratory experiments that coensiled maize and sugar beets increased biogas production while supplementing macro- and micronutrients instead of adding manure. Böttcher et al. [33] investigated that an addition of less than 20% sugar beet silage (volatile solid based) increased the methane yield. However, at a share of more than 50%, the process became unstable. Viscosity problems, which are very common for monodigestion of energy crops, were minimized by sugar beet silage as co-feedstock [5].

In soil sciences, readily available carbohydrates are known to increase soil organic matter decomposition as they spur the overall microbial activity. This improvement of organic matter degradation occurs due to the higher availability of energy released from the freshly decomposed organic matter to the microorganisms and regarded as “priming effect” [34]. This can also be applicable for AD of fibrous substrates. While investigating different mixtures of sugar beet silage with maize and grass silage, respectively, our study focused on the hypothesis that the easily degradable carbohydrates of the sugar beet silage will improve the anaerobic digestion of maize and grass silage. Based on the “priming effect,” co-digestion of sugar beet silage shall positively affect the digestion and increase the methane yield from the mixtures. Furthermore, the AD performances of maize silage and grass silage, respectively, are expected to be stabilized by addition of sugar beet silage. These effects are expected to be more prominent for fibre-rich grass silage than for maize silage.

To test these hypotheses, anaerobic co-digestions of sugar beet silage with maize silage and grass silage were investigated in two separate experiments using four parallel CSTRs (continuously stirred tank reactors). Different ratios of sugar beet silage were tested in order to examine the following: (i) process stability in terms of pH and the ratio of total volatile fatty acid (TVFA) to total alkaline capacity (TAC) with respect to VFA concentration, (ii) reactor performance expressed as biogas and methane production, and (iii) volatile solids (VS) degradation. Finally, methane yields obtained from the experiment were compared to the theoretical yield in order to elucidate synergistic effects of sugar beet silage co-digestion.

## 2. Materials and Methods

### 2.1. Setup and Operation of CSTRs

The experiments were conducted in four CSTRs, with operating volume of 10 L, and run at mesophilic temperature of 40°C. The cylindrical, double-layered biogas reactors made of stainless steel were built in the mechanical workshop of the Ulm University under supervision of author's institute. A thermostat (JULABO GmbH, Seelbach, Germany) heated the water for temperature adjustment to 42°C which was constantly circulating in the reactor's jacket.

Feedstocks were carried into the reactor by screw conveyor placed on the base of feedstock storage tube. The operating unit allowed subdivision of each rotation into 12 impulses (1 impulse = 30°). Digital card processed under LabVIEW was sending fixed number of impulses to the feeding system at given time interval. In the present experiment, screw conveyor revolutions were set individually for each mixture by the operating unit. The feedstock inputs were realized automatically on an hourly basis. Mixing of sugar beet silage with maize silage produces high surface tension inside the feedstock storage tubes. Thus, surfaces of tubes were regularly cleaned for good performance [23]. Homogeneous mixture inside the reactor was achieved by steering every 10 minutes for 5 minutes at 80 rpm with paddle mixers. An unheated, air tight container was used to store the effluent passing out through the upper part of the reactor.

### 2.2. Feedstocks and Inoculum

Two experiments were carried out subsequently. In experiment one (M : S), four CSTRs were run with mixtures of maize silage (M) and sugar beet silage (S) in a ratio of (based on VS) 1 : 0 (CFM1) (monofermentation of maize silage), 6 : 1 (CFM2), 3 : 1 (CFM3), and 1 : 3 (CFM4). The same VS ratios were used for experiment two (G : S) for mixtures of grass silage (G) and sugar beet silage (S), using similar nomenclature for the reactors CFG1 (monofermentation of grass silage), CFG2, CFG3, and CFG4, respectively. Sugar beet silage originated from Raiffeisen Warengenossenschaft Emsland-Süd eG, Lünne, Germany. It was covered and stored at 5°C. Maize silage and grass silage were obtained from a biogas plant in Gögglingen, Germany. Substrate characteristics are depicted in Table 1. Maize was collected at the period of late ripening and harvesting time of grass was late summer. Maize and grass were chopped immediately and ensiled without the use of any additive. This type of grass is also called haylage because of low water content. For better mixing behaviour, both feedstocks were cut into 10–15 mm long pieces.

In both experiments, the C/N ratio of feedstocks was between 15 and 34 (Table 2), thus in a range that does not influence methanogens by toxic levels of ammonia [35]. The C/N ratio of digestate was within the range as reported by Amon et al. [36] (Table 2), also indicating a good anaerobic digestion process.

The inoculum originated from a mesophilic (40°C) full-scale “ring-in-ring” biogas plant (Ulm-Gögglingen, Germany) fed with pig manure (30%), maize silage (56%), and grass silage (14%). Inoculum was used to fulfil the basic nutrient requirements for anaerobic microorganism. It was filtered and kept at 40°C under anaerobic conditions for several days in order to minimize its methane production [37]. Before experimental start, an addition of feedstock started at low OLR (organic loading rate) of about 0.5 kgVS m^−3^ day^−1^ till constant methane production rates (data not shown). After reaching process stability in terms of biogas yields and alkalinity ratios (0.05 ± 0.01), the analysed period of the experiments began. The OLR was then increased to 1.5 kgVS m^−3^ day^−1^ for both experiments. The daily feeding amount was given based on fresh material calculated from volatile fraction of respective experiments (Table 2). The fermentation process was evaluated for a period of 50 days for both experiments.

### 2.3. Analytical Methods

Biogas volumes were assessed online by Milligascounters (MGC-10, Ritter GmbH, Bochum, Germany) and normed volumes (l_N_) were given at standard pressure and temperature (at 1.013 bar, 0°C, and 0% RH). Methane concentrations were measured (relative error: ±2% of the measured value) by using infrared sensors manufactured by Bluesens GmbH (BCP-CH4, Herten, Germany). Methane sensors were calibrated with calibration gas consisting of 60% CH_4_ and 40% N_2_. pH values were measured twice a week by pH electrodes (SensoLyt® SE, WTW GmbH, Weilheim, Germany). Temperature sensors (10k Thermistor, UP Umweltanalytische Produkte GmbH, Cottbus, Germany) were used for measuring the reactor temperature and then logged hourly with a data logger (DL2e, Delta-T, Burwell, UK).

Other process parameters like total solids (TS), VS, ratio of total volatile fatty acid (TVFA) to total alkaline capacity (TAC) (alkalinity ratio), and carbon (C) to nitrogen (N) ratio of the slurry were measured weekly. The measurements of TS and VS were carried out according to APHA standard methods 2540B and 2540E [38]. Volatile fatty acids (ethanol, acetate, propionate, and butyrate) were determined with a gas chromatograph (CP 9001, Chrompack, Rodgau, Germany) using a 0.32 mm diameter capillary column with 30 m length and helium carrier gas with a flow rate of 1 mL min^−1^. The alkalinity ratio was measured by automatic titration (Dosimat 665, Metrohm, Herisau, Switzerland) with 1 M HCL to endpoints of pH 5.0 and pH 4.3 [39]. Total carbon and nitrogen were analysed by C/N analyser (TrueSpec, LECO Instrumente GmbH, Mönchengladbach, Germany). Ash-free neutral detergent fibre (NDFom), ash-free acid detergent fibre (ADFom), acid detergent lignin (ADL), and relevant characteristics (crude protein, crude fat, starch, sugar, and crude fibre) were determined by the Institute for Oil and Environment (LUFA Nord-West, Oldenburg, Germany). Structural carbohydrates (cellulose, hemicellulose, and lignin) were obtained from NDFom, ADFom, and ADL. Organic loading rates (OLR, kgVS m^−3^ day^−1^) were calculated by dividing the daily feedstocks inputs (kgVS day^−1^) into the reactor's operation volume. Specific biogas yields (l_N_ kg^−1^VS) were obtained by dividing the total biogas volume (l_N_) obtained during the respective days by the total feedstock input (kgVS).

### 2.4. Biodegradability

VS concentration in the reactor effluent was inhomogeneous due to high fibrous feedstock. Moreover, frequent sampling also altered the amount of effluent. So, the VS degradation was calculated based on VS contents in reactors and VS contents of feedstocks according to Koch et al. [10] assuming constant nondegradable material.

(1) $$\begin{array}{c} VS\;\;degradation\left(\;\%\;\right)=1-\frac{{VS}_{reactor}\cdot \left(1-{VS}_{feed}\right)}{{VS}_{feed}\cdot \left(1-{VS}_{reactor}\right)}, \end{array}$$

where VS_reactor_ refers to the VS content of the reactor (% of TS) and VS_feed_ gives VS contents of the feedstocks in % of TS.

### 2.5. Synergistic Effects and Theoretical Yield

Synergistic effects can occur in co-digestion of different components, representing positive influence of each feedstock in the mixture. In this study, possible synergetic effects were calculated by (2) [40].

(2) Synergistic  effects=Experimental  methane  yieldEMYTheoretical  methane  yieldTMYM.

The “Experimental methane yield” was the yield produced in co-digestion mixture of the according reactor, calculated from last 20 days. The TMY_M_ considered theoretical methane yield for sole feedstock multiplied by relative VS content of each substrate in the mixture.

(3) $$\begin{array}{c} {TMY}_{M}\left(\;l_{N}/kgVS\;\right)=\frac{\left({TMY}_{F}\cdot \alpha +{TMY}_{S}\cdot \beta \right)}{\alpha +\beta }, \end{array}$$

where TMY_F_ and *α* referred to theoretical methane yield (l_N_ kg^−1^VS) and VS fraction for maize silage or grass silage, respectively. TMY_S_ and *β* referred to the one for sugar beet silage.

In this study, the values of TMY_F_ for maize silage and grass silage were considered equivalent to specific methane yields from their respective monofermentation (CFM1 and CFG1) as equilibrium condition was achieved with saturated yield for the last 20 days. On the other hand, monofermentation of sugar beet silage (VS ratio 0 : 1) is not reliable for several reasons mentioned above and the results can be biased due to foaming and excessive CO_2_ evolution after substrate input. Therefore, the calculation of theoretical methane yields was based on a comprehensive analysis of the constituents of the used sugar beet silage (Table 1) and was done according to Buswell formulae [41–43]. Methane yield was primarily obtained for organic substrates like VFA (as C_2_H_4_O_2_), lipids (as C_57_H_104_O_6_), protein (as C_5_H_7_NO_2_), carbohydrates (as C_6_H_10_O_5_), and lignin (as C_10_H_13_O_3_). According to its organic composition (Table 1), theoretical methane yield (TMY_S_) of sugar beet silage was 354 l_N_ kg^−1^VS (see (4)). The calculated yield agrees well with the value given of 360 l_N_ kg^−1^VS by KTBL [2].

(4) $$\begin{array}{c} {TMY}_{S}\left(\;l_{N}/kgVS\;\right)=\frac{\left(374\;\;VFA+496\;\;Protein+1014\;\;Lipids+415\;\;Carbohydrates+728\;\;Lignin\right)}{100}. \end{array}$$

### 2.6. Statistical Analysis

Statistical data analysis was carried out with the software R (statistics version 3.2.0). Specific biogas and methane production rates during both experiments were evaluated by descriptive statistics. Furthermore, normal distribution was checked by Shapiro-Wilk test [44]. Mean, median, and variance have been determined for parametric and nonparametric tests. Parametric data (M : S) and nonparametric data (G : S) were further analysed with one-way ANOVA and Kruskal-Wallis test, respectively, to find significant differences among the reactors [45]. Kruskal-Wallis post hoc test was used for multiple comparisons among the reactors for G : S experiment. The significant level was set at *p* < 0.05.

## 3. Results and Discussion

### 3.1. Process Performance

#### 3.1.1. pH and TVFA/TAC Ratio

**Figure 1:**
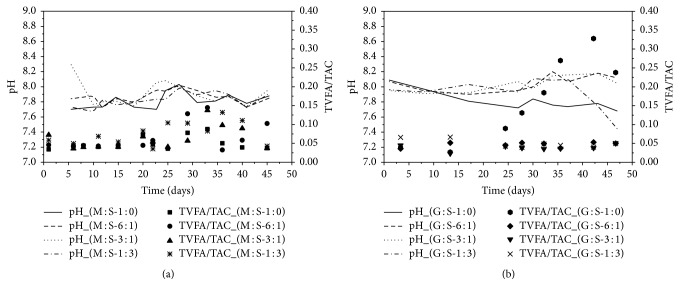
pH (lines) and TVFA/TAC (dots) during fermentation process of M : S (a) and G : S (b).

During fermentation of the experiment one (M : S), pH value was in the range between 7.7 and 8.03 (Figure 1(a)) indicating a stable AD process [46]. Also, the reactor CFM4 (M : S-1 : 3), with the highest share of sugar beet silage, showed an optimal pH value. The TVFA/TAC ratio was below 0.3 in all reactors, indicating process stability and implying sufficient buffer components without any acidification risk [11].

In case of experiment two (G : S), the anaerobic digestion (AD) showed a more differentiated picture (Figure 1(b)); pH value in the reactor CFG1 (G : S-1 : 0) was decreasing while TVFA/TAC values increased up to 0.34. As a consequence, methane production decreased. Thus, monofermentation of grass silage may become unstable due to lower buffering capacity [11]. Sugar beet silage obviously stabilized the process with respect to pH as well as TVFA/TAC values in reactors CFG2 and CFG3. However, in reactor CFG4, with the highest sugar beet silage ratio, pH value declined from 8.2 to 7.3 at the end of the experimental period, implicating possible process disturbance in such feeding regime [23].

#### 3.1.2. Total Solids Accumulation

**Figure 2:**
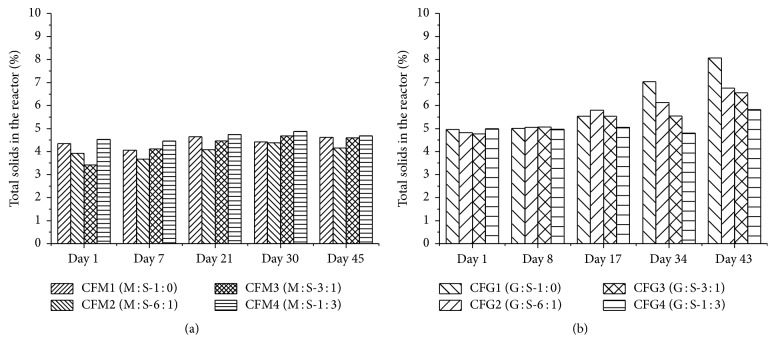
Effect of sugar beet silage on total solid accumulation during the fermentation process: M : S (a) and G : S (b).

During experiment one (M : S), the TS accumulation did not occur and the TS remained in the range of initial inoculum (4.7%  ±  0.2, *n* = 8). Independent of the M : S mixtures, the TS value within all reactors varied from 4.5% to 5.0% (Figure 2(a)). On the other hand, grass silage is known as buoyant biomass forming floating mass on the slurry surface, which can gradually produce indigestible scum [47, 48]. Such effect was realized in reactor with monofermentation of grass silage (CFG1) where TS accumulation occurred after 17 days and ended at 8.0% TS by the end of the experiment (Figure 2(b)). The TS accumulation was much lower with higher share of sugar beet silage in CFG3 and CFG4. Its positive effects on the digestion process can even be seen in CFG2 (G : S-6 : 1) with low amount of sugar beet silage in the substrate mixture which showed a final TS value of 6.7%.

In our experiments, even minor sugar beet addition to such fibrous feedstock was found to improve the homogenization and reactor functioning. Thus, accumulation of TS was avoided with addition of sugar beet silage.

#### 3.1.3. VFAs Concentration

Volatile fatty acids (VFAs) like acetate, propionate, and butyrate are mainly produced in degradation of complex organic polymers during hydrolysis and acidogenic stages [49]. VFAs concentrations were below 0.4 g L^−1^ during experiment one (M : S) (data not shown), implying no inhibition during the fermentation period. This is referred to as an optimal range for anaerobic digestion of maize and sugar beet silage [23, 50]. The average ethanol concentration was about 0.02 g L^−1^ (±0.01, *n* = 7) in all reactors, which was considered low in spite of using sugar beet silage. Ethanol production was slightly increased in CFG4 at day 17, with concentrations of 0.32 g L^−1^. Afterwards, it declined again and the average value for ethanol concentration was 0.01 g L^−1^ among all reactors.

**Figure 3:**
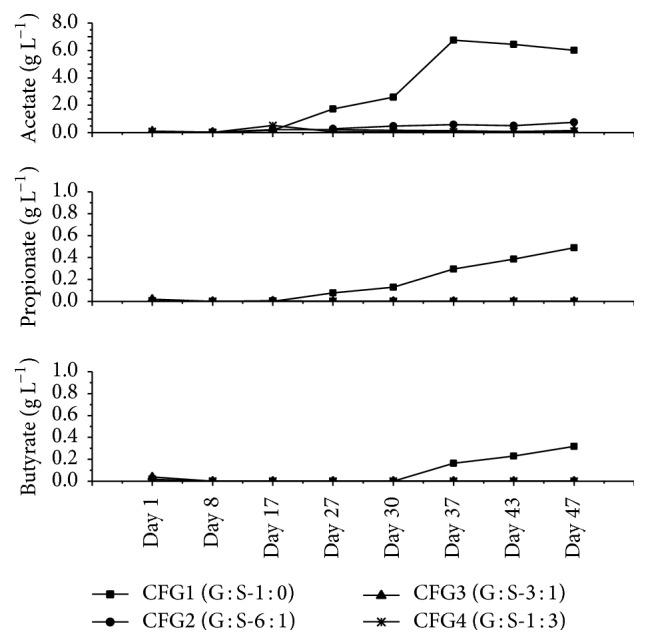
Individual VFAs (acetate, propionate, and butyrate) concentration in each reactor during co-fermentation of grass silage (mixture ratios for each reactor are based on volatile solids).

**Figure 4:**
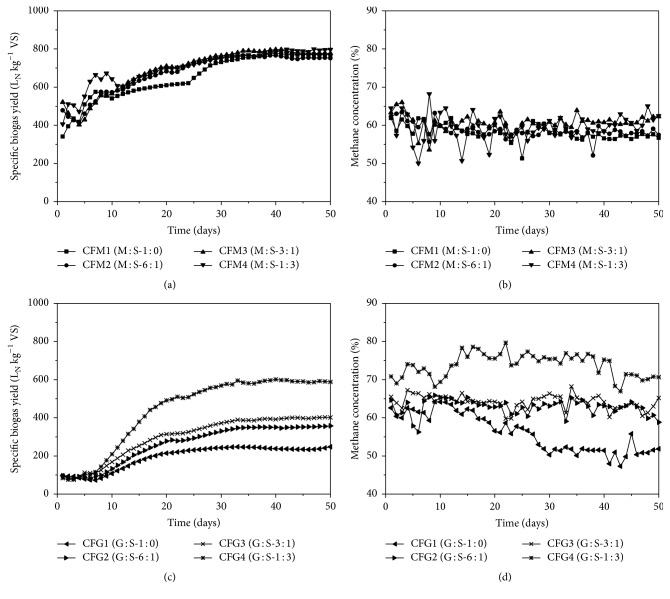
Specific biogas yield and methane concentration during the fermentation period: (a), (b) experiment M : S; (c), (d) experiment G : S, respectively.

In experiment two (G : S), the VFAs concentrations (Figure 3) were stable in all reactors, except CFG1 (G : S-1 : 0). In the reactor CFG1, acetate, propionate, and butyrate concentrations at the end of the experiment were as high as 6.01 g L^−1^, 0.49 g L^−1^, and 0.31 g L^−1^, respectively (Figure 3). According to Drosg [46], stable digestion is presumed up to 4.3 g L^−1^ of total VFAs concentration. Similar results were found by Pakarinen et al. [51], where biogas yield was decreased due to higher amount of acetate, propionate, and butyrate, respectively. According to Kus and Wiesmann [52], propionate degradation is being inhibited due to acetate accumulation. Increasing accumulation of VFAs reflects an imbalance associated with a pH drop and reduced buffer capacity [53], which was also found in CFG1 in experiment two. The accumulation of undigested material can also lead to an increase of propionate and butyrate concentrations, which inhibits methanogens [4, 5], thus decreasing methane concentrations (see Figure 4(d)).

On the contrary, acetate concentrations were lower than these values (0.76 g L^−1^, 0.15 g L^−1^, and 0.11 g L^−1^) in reactors CFG2, CFG3, and CFG4, respectively, where sugar beet silage was used as co-feedstock. Furthermore, the concentrations of propionate and butyrate were within the range considered as optimal for fermentation process [54]. Addition of sugar beet silage as co-feedstock to grass silage resulted in lower accumulations of VFAs. When other co-feedstocks like pig manure were added at different ratios to grass silage [11], VFA concentrations were higher compared to this experiment.

### 3.2. Biogas Yield and Methane Production Rate

Similar biogas yields produced among all reactors during experiment one (M : S) (Figure 4(a)) indicate no functional effect of sugar beet silage. Specific biogas yields in CFM1 (1 : 0) and CFM2 (6 : 1) were 769 l_N_ kg^−1^VS and 751 l_N_ kg^−1^VS, respectively. A share of sugar beet silage at 3 : 1 (CFM3) and 1 : 3 (CFM4) only slightly increased the biogas yield from 777 l_N_ kg^−1^VS to 797 l_N_ kg^−1^VS. Therefore, yields obtained in the M : S experiment were typical for AD reactors with low OLR when utilizing readily digestible material, that is, maize silage [55]. Furthermore, specific methane yield was similar in CFM1 and CFM2 and increased slightly with higher amount of sugar beet addition (CFM3: 484 l_N_ kg^−1^VS, CFM4: 498 l_N_ kg^−1^VS) (data not shown). All reactors produced biogas at average daily methane concentration of 59% (±3, *n* = 200).

In case of the G : S experiment, the effects of sugar beet silage on biogas yield and methane concentrations were quite obvious (Figures 4(c) and 4(d)). Maximum biogas yield of 588 l_N_ kg^−1^VS was obtained in CFG4 whereas biogas yield in CFG1 was as low as 247 l_N_ kg^−1^VS. In comparison to CFG1, biogas yield increased by 44% (357 l_N_ kg^−1^VS) with only small sugar beet addition in CFG2 (6 : 1). Higher addition of sugar beet silage in CFG3 (3 : 1) further increased the biogas yield by about 62%. Specific methane yield was also enhanced with increasing share of sugar beet silage. Maximum methane yield of 415 l_N_ kg^−1^VS (data not shown) was produced in CFG4, with average daily methane concentrations up to 74% (±3, *n* = 50) (Figure 4(d)).

In comparison to experiment one (M : S), the biogas yield was low in experiment two (G : S). Maize silage contains lower lignin content compared to grass silage, although both feedstocks had the same cellulose/lignin ratio. According to McKendry [56], biomass high in cellulose can still produce lower yields in biochemical conversion if the lignin contents are simultaneously high.

Average daily methane concentration was remarkably increased in CFG4 (Figure 4(d)), also compared to CFM4 (Figure 4(b)). The reason for higher methane concentration in CFG4 can be attributed to formation of stable biofilms on the grass silage enhancing methane production [57, 58]. The effective metabolism of microbial community could lead to higher methane concentration in this case. In addition, the microbial community assembled in biofilms avoided excessive CO_2_ production that can occur during rapid degradation of sugar beet silage components (sugars, alcohols, and carboxylic acids). This can intensify the production of organic acids and lead to fluctuating methane concentration as observed while utilizing higher amounts of sugar beet silage [59] also in co-fermentation with maize silage [33]. Similar behaviour was found in our case in CFM4 (M : S-1 : 3) but was not the case in CFG4 (G : S-1 : 3). Presumable influence of biofilms in CFG4 resulted in higher methane concentration.

Biogas yield became stable in both experiments from day 30 on (Figures 4(a) and 4(c)). Due to this fact, specific biogas and methane production rate was evaluated for the last 20 days in both experiments. For the M:S mixtures, similar production trends were found among all reactors (*n* = 20; *p* > 0.05, Table 3). In experiment two (G : S), specific biogas as well as methane production rates increased significantly (*n* = 20, *p* < 0.05) when sugar beet silage was added to grass silage. The lowest specific biogas production rate of 0.27 l_N_ kg^−1^VS h^−1^ (±0.09, *n* = 20) was found in CFG1 and increased towards 0.68 l_N_ kg^−1^VS h^−1^ (±0.31, *n* = 20) in CFG4 (Table 3). Low moisture content in grass silage reduced the methane production rate, since methanogenesis can be inhibited in digestion of feedstock with higher TS content. Such effect was observed in CFG1 with monofermentation of grass silage even though intensive mixing was applied [60]. Sugar beet silage addition to grass silage prevented such process disturbance and resulted in increased methane production. Several properties of the sugar beet silage may contribute: (i) lower TS of sugar beet silage, (ii) high amounts of easily degradable components, which led to a better homogenization of the reactor content, and (iii) improved grass silage degradation. Still, no significant differences in specific biogas production rate were found between CFG2 and CFG3 (*p* > 0.05). In case of grass silage, process efficiency increased already by adding sugar beet silage at comparatively low sugar beet content with VS ratio of 6 : 1 (G : S). Specific methane production rate of 0.14 l_N_ kg^−1^VS h^−1^ (±0.05, *n* = 20) in CFG1 was more than tripled to 0.50 l_N_ kg^−1^VS h^−1^ (±0.24, *n* = 20) in CFG4 (Table 3) at the same VS loading.

Also, other techniques such as leach bed reactors can produce high methane yields from the leachates obtained by hydrolytic pretreatments of lignocellulosic biomass [14, 15]. Still, it has to be underlined that the investigated effects of comparatively low addition of sugar beet silage to fibrous energy crops can be applied at comparatively little changes and low costs in already existing biogas plants.

### 3.3. Degradation of Volatile Solids

Differences in VS degradation were found in this study reflecting process efficiency. Degradation level was high in fermentation of maize silage compared with the experiment investigating grass silage mixtures. VS degradation was about 80% in all CFM1, CFM2, CFM3 and slightly higher in CFM4 with 84%. These degradation levels are usual for energy crops [61]. It stays in contrast to the G : S experiment, where VS degradation improved notably in mixtures with sugar beet silage. Monodigestion of grass silage (CFG1) showed VS degradation as low as 22%. Grass silage was rich in fibres as indicated by high TS (57%) and low VS ratio (74%) which can explain the lower VS degradation in CFG1. Literature implies that VS degradation of grass silage can be higher. Koch et al. [10] investigated AD of grass silage in loop reactors and reported degradation of 60% for grass silage containing 50% TS and 91% VS. In comparison with CFG1, the degradation had increased to about 36% by adding 14% of sugar beet silage to CFG2. More surprisingly, VS degradation did not increase further with doubling the sugar beet share in CFG3 (G : S mixture of 3 : 1). The CFG4 reactor with 75% sugar beet silage showed the highest VS degradation of 54%.

### 3.4. Synergistic Effects of Sugar Beet Silage on Methane Production

Co-fermentation of sugar beet silage and grass silage showed positive effect on biogas yield (Figure 4(c)) and was tested also regarding the methane yield. Additional methane yield from co-digestion of grass silage and sugar beet silage overweighted the yield of individual feedstock, thus showing synergism of both substrates. Synergistic effects of tested substrates are mostly evaluated by biochemical methane potential (BMP) assay [40] which was not performed in this study. However, synergistic effects can be evaluated in the continuous process using (2).

The results indicated that the sugar beet co-digestion had slightly positive effect during experiment one (M : S), while the mixtures in experiment two (G : S) showed strong effects independently of the amount of sugar beet silage (Table 4).

Addition of sugar beet silage provided acids to degrade partially the fibrous grass silage which may increase the hydrolysis rate and improved degradation of cellulose which is the major component of grass silage (Table 1) [11]. The biodegradability of grass silage was enhanced by sugar beet silage addition even at low share in CFG2, supporting the “priming effect” concept [34].

These results emphasize the preferential use of sugar beet silage for co-fermentation with fibre-rich substrates like grass silage. Furthermore, high share of sugar beet silage as a co-feedstock led to yields, which were even higher than in other types of co-fermentation. For example, a yield of 125 L CH_4_ kg^−1^VS was obtained from co-digestion of dairy manure and switchgrass [25] and 215.2 L CH_4_ kg^−1^VS was gained from animal manure and maize silage [24].

## 4. Conclusions

The co-fermentation of sugar beet silage with fibrous substrates brings easy-to-digest compounds to the microbial community. This improves the overall degradation and biogas production as shown in the reactors cofermenting grass silage with low shares of sugar beet silage, thus confirming the study hypothesis. Moreover, the concept of “priming effect” known from terrestrial ecosystems can also be extended to anaerobic degradation of organic matter.

Although little effects were realized in co-fermentation with maize silage, easily degradable components of sugar beet silage led to increase of digestibility and higher methane yields in grass silage reactors. Moreover, even small addition of sugar beet silage was sufficient for such effect. Co-fermentation of carbohydrate-rich feedstocks (e.g., sugar beet silage) together with fibre-rich biomass (e.g., grass silage) improves obviously the anaerobic degradation of the latter.

In many countries, there are large amounts of fibre-rich substrates like grass silage, straw, material from landscaping, urban greening, and so forth, which are often left aside from regular use in AD. The described addition of sugar beet silage at even low ratios will be a low-cost option to include the mentioned substrates in production of renewable energy.

## Figures and Tables

**Table 1:** Characteristics of feedstock and inoculum.

Parameter	Inoculum	S	M	G
pH	7.7	3.5	5.0	5.1
TS (%)^a^	5.2	13.0	35.7	57.0
VS (%)^a^	3.2	11.3	28.5	42.2
VS/TS (%)	61.3	86.8	80.0	74.0
C (%)^b^	39.7	37.7	46.3	42.1
N (%)^b^	3.6	1.1	1.4	2.8
C/N	11.2	34.0	33.2	15.0
VFA as C_2_H_4_O_2_ (g kg^−1^)^b^	ND	35.0	ND	ND
Lignin (g kg^−1^)^b^	ND	<9.0	22.0	32.0
Cellulose (g kg^−1^)^b^	ND	64.0	160.0	249.0
Hemicellulose (g kg^−1^)^b^	ND	76.0	158.0	112.0
Crude protein (g kg^−1^)^b^	ND	81.0	79.0	157.0
Crude fat (g kg^−1^)^b^	ND	8.0	29.0	39.0
Starch (g kg^−1^)^b^	ND	74.0	397.0	5.0
Sugar (g kg^−1^)^b^	ND	32.0	<5.0	6.0

ND: not determined; S: sugar beet silage; M: maize silage; G: grass silage.

^a^Fresh mass of sample; ^b^total solid mass of sample.

**Table 2:** Reactors with feedstock ratio and volatile solid contents. C/N ratio of feedstock in mixtures and in digestate during fermentation period.

	VS ratio	FM (%)	C/N ratio of feedstocks in mixtures	C/N ratio in digestate
Experiment one (M : S)				
CFM1	1 : 0	100/0	33.1	11.1 ± 0.3
CFM2	6 : 1	70/30	33.2	11.1 ± 0.2
CFM3	3 : 1	54/46	33.3	11.3 ± 0.3
CFM4	1 : 3	12/88	34.0	11.4 ± 0.6
Experiment two (G : S)				
CFG1	1 : 0	100/0	15.0	12.3 ± 0.6
CFG2	6 : 1	62/38	16.1	11.6 ± 0.4
CFG3	3 : 1	45/55	17.0	11.5 ± 0.5
CFG4	1 : 3	8/92	24.7	11.2 ± 0.5

FM: fresh masses; VS: volatile solids.

**Table 3:** Specific biogas and methane production rates evaluated for both experiments for the last 20 days.

Reactors	*n*	sBPR (l_N_ kg^−1^VS h^−1^)	sMPR (l_N_ kg^−1^VS h^−1^)
M : S			
CFM1 (M : S-1 : 0)	20	0.90 ± 0.23^a^	0.51 ± 0.14^a^
CFM2 (M : S-6 : 1)		0.81 ± 0.24^a^	0.47 ± 0.14^a^
CFM3 (M : S-3 : 1)		0.86 ± 0.26^a^	0.52 ± 0.16^a^
CFM4 (M : S-1 : 3)		0.93 ± 0.27^a^	0.55 ± 0.15^a^
G : S			
CFG1 (G : S-1 : 0)	20	0.27 ± 0.09^a^	0.14 ± 0.05^a^
CFG2 (G : S-6 : 1)		0.44 ± 0.14^b^	0.27 ± 0.09^b^
CFG3 (G : S-3 : 1)		0.49 ± 0.16^b^	0.31 ± 0.10^b,c^
CFG4 (G : S-1 : 3)		0.68 ± 0.31^c^	0.50 ± 0.24^c^

sBPR: specific biogas production rate; sMPR: specific methane production rate; different letters in parenthesis show significant differences among reactors for each experiment at *p* < 0.05.

**Table 4:** Experimental methane yield (EMY) and theoretical methane yield (TMY) for all experiments from days 30 to 50. Synergistic effects of co-digestion are indicated by EMY/TMY ratio fairly above 1.

Reactors	EMY (l_N_ kg^−1^VS)	TMY (l_N_ kg^−1^VS)	Synergistic effects (EMY/TMY)
M : S			
CFM1	475 ± 70	475	1,00
CFM2	437 ± 96	458	0,95
CFM3	485 ± 93	445	1,09
CFM4	520 ± 13	384	1,35
G : S			
CFG1	131 ± 51	131	1.00
CFG2	252 ± 52	163	1.55
CFG3	288 ± 64	187	1.54
CFG4	459 ± 17	298	1.54
